# SARS-CoV-2 variant B.1.1.7 susceptibility and infectiousness of children and adults deduced from investigations of childcare centre outbreaks, Germany, 2021

**DOI:** 10.2807/1560-7917.ES.2021.26.21.2100433

**Published:** 2021-05-27

**Authors:** Anna Loenenbach, Inessa Markus, Ann-Sophie Lehfeld, Matthias an der Heiden, Walter Haas, Maya Kiegele, André Ponzi, Barbara Unger-Goldinger, Cornelius Weidenauer, Helen Schlosser, Alexander Beile, Udo Buchholz

**Affiliations:** 1 Department for Infectious Disease Epidemiology, Robert Koch-Institute, Berlin, Germany; 2 These authors contributed equally to this article and share first authorship; 3 Federal Office of Administration, Cologne, Germany; 4 Local Health Authority Bergstraße/Hesse, Heppenheim, Germany

**Keywords:** COVID-19, SARS-CoV-2, VOC, B.1.1.7, kindergarten, outbreak, secondary attack rate, childcare centre, susceptibility, infectiousness

## Abstract

We investigated three SARS-CoV-2 variant B.1.1.7 childcare centre and related household outbreaks. Despite group cohorting, cases occurred in almost all groups, i.e. also among persons without close contact. Children’s secondary attack rates (SAR) were similar to adults (childcare centres: 23% vs 30%; p = 0.15; households: 32% vs 39%; p = 0.27); child- and adult-induced household outbreaks also led to similar SAR. With the advent of B.1.1.7, susceptibility and infectiousness of children and adults seem to converge. Public health measures should be revisited accordingly.

In December 2020, the severe acute respiratory syndrome coronavirus 2 (SARS-CoV-2) ‘variant of concern’ (VOC) B.1.1.7 (N501Y.V1) began to circulate in Germany. As at 3 March 2021, approximately 40% of all randomly selected swabs of all coronavirus disease (COVID-19) cases tested positive for the B.1.1.7 variant [1]. While several studies have presented evidence for the increased transmissibility of B.1.1.7 [2-4] there is limited evidence on the susceptibility and infectiousness of children, particularly for those of preschool age [5,6]. Here we describe the investigation of three childcare centre outbreaks caused by the B.1.1.7 variant and our assessment of secondary attack rates (SAR) within the centres and associated households.

## Outbreaks in childcare centres and investigations conducted

In January–February 2021, three B.1.1.7 variant childcare centre outbreaks occurred almost simultaneously in a district of one local health authority (LHA) in the State of Hesse. Together with the related household situations these outbreaks gave us the opportunity to assess: (i) if high-risk contact definitions (within 1.5 m distance for at least 15 min) still hold; (ii) if there are indications of increased susceptibility of preschool children in comparison to adults; and (iii) the infectiousness of preschool children, in comparison to adults in household settings.

A joint outbreak investigation team from the LHA and the Robert Koch Institute (RKI, national public health institute) in Berlin, investigated the outbreaks and collected information regarding structural, organisational and infection control measures in the facilities. The childcare centres cared for between 26 and 49 children, aged from 1 to 6 years. Two to five staff took care of the children, cohorted in two to four groups. All childcare centres had an additional daycare group for children up to 3 years, which was structurally separated within the buildings.

In response to the COVID-19 pandemic, all three childcare centres had the following hygiene and infection control measures in place: (i) cohort grouping with a reduced number of children and designated staff, and access to separate bathroom areas, (ii) playing outside only within same groups in assigned playground areas, (iii) staff wearing masks at least outside of the group rooms, (iv) parents not allowed to enter the building and mandatory mask-wearing during children’s drop off and pick up, and (v) conducting meetings digitally with staff and/or parents, if possible.

### Secondary attack rates in childcare centres

Following the outbreaks’ detection, the outbreak investigation team visited the facilities and conducted interviews with childcare centres’ management and primary and/or first secondary cases. Comprehensive testing for SARS-CoV-2 was offered to all children and personnel at the facilities. All contact persons were followed up daily for symptoms via telephone calls by public health workers at the LHA. Any person becoming symptomatic was tested by PCR. We considered the outbreak as over, when over a time period of 14 days no new cases occurred. Those with a positive PCR test within this time frame were considered as secondary cases. Centre contact persons testing negative or in the absence of a test (n = 13, 3 and 15, in childcare centres 1, 2 and 3, respectively) and without symptoms, were counted as non-cases. We defined as a close contact person anyone with an encounter to the primary case (PC) of ≥ 15 min within a distance of < 1.5 m. All other persons in the childcare centres were defined as non-close contacts.

The overall SAR in the three childcare centres were 37% (95% confidence interval (CI): 26–49), 27% (95% CI: 16–42) and 17% (95% CI: 9–28) (Table 1). In all three outbreaks the likely PCs were adults. None of them reported respiratory symptoms as the initial symptoms at illness onset, but rather general, unspecific symptoms, such as fatigue, headache, back pain or exhaustion. In all three childcare centres, 80% to 88% of secondary cases occurred within an interval of 7 days (Table 1).

**Table 1:** Secondary attack rates of SARS-CoV-2 B.1.1.7 variant infections in sub-groups, by type of contact and by age in three childcare centre outbreaks, Hesse, Germany, January–February 2021

	Childcare centre 1			Childcare centre 2			Childcare centre 3		
	n/N	SAR (%)	95% CI	n/N	SAR (%)	95% CI	n/N	SAR (%)	95% CI
Overall SAR	25/68	37	26–49	12/44	27	16–42	10/59	17	9–28
7 days clustering^a^	22/25	88	70–96	10/12	83	55–95	8/10	80	49–94
**Group**									
Group 1 (with PC)	8/15	53	30–75	3/9	33	12–65	6/18	33	16–56
Group 2	7/11	64	35–85	6/15	40	20–64	3/20	15	5–36
Group 3	2/9	22	6–55	1/7	14	3–51	NA	–	–
Group 4	5/16	31	14–56	NA	–	–	NA	–	–
Daycare	2/12	17	5–45	0/7	0	0–35	1/15	7	1–30
Staff	1/5	20	4–62	2/6	33	10–70	0/6	0	0–39
**Type of contact**									
Close contact	8/15	53	30–75	3/9	33	12–65	9/41	22	12–37
Non-close contact	17/53	32	21–45	9/35	26	14–42	1/18	6	1–26
**Age category**									
Children	15/49	31	20–45	7/26	27	14–46	6/36	17	8–32
Adults	10/19	53	32–73	5/18	28	12–51	4/23	17	7–37

CI: confidence interval; n: number; N: total number; NA: not applicable; PC: primary case; SAR: secondary attack rates; SARS-CoV-2: severe acute respiratory syndrome coronavirus 2.

^a^ Clustering of secondary cases within an interval of 7 days. For asymptomatic persons (childcare centre 1: n = 7; childcare centre 2: n = 5; childcare centre 3: n = 2) the day of testing was assigned.

Eleven of all twelve cohorted groups had secondary cases. SAR for close contact persons were 53% (95% CI: 30–75), 33% (95% CI: 12–65) and 22% (95% CI: 12–37), and among non-close contacts 32% (95% CI: 21–45), 26% (95% CI: 14–42) and 6% (95% CI: 1–26). SAR for children were 31% (95% CI: 20–45), 27% (95% CI: 14–46) and 17% (95% CI: 8–32), and among adults 53% (95% CI: 32–73), 28% (95% CI: 12–51) and 17% (95% CI: 7–37) (Table 1).

### Secondary attack rates in households

Households were not tested systematically. However, we offered contact persons a PCR test shortly after the PC tested positive. Those who tested positive within the two-week quarantine were counted as cases. We contacted households to monitor symptom onset dates and tested symptomatic persons. Household contact persons testing negative or, in the absence of a test (19/92; 21%), without symptoms, were counted as non-cases. After exclusion of single person households (n = 2) and households with another possible PC (n = 6), we included 38 households with 92 contact persons.

Pooled household SAR was 37% (95% CI: 28–47; Table 2). If the household contact person was a child (32%; 95% CI: 18–51), SAR was lower but not significantly different compared with adult household contact persons (39%; 95% CI: 28–51). SAR in households were higher when the PC was a child (39%; 95% CI: 28–52) vs adult PC (33%; 95% CI: 20–50), although this difference was not statistically significant.

**Table 2:** Secondary household attack rates of SARS-CoV-2 B.1.1.7 variant infections among household contacts of primary cases from three childcare centre outbreaks, Hesse, January–February 2021

Household situation	n/N	SAR in %	95% CI	RR	95% CI
**All**	34/92	37	28–47	NA	NA
Household CP is a child	9/28	32	18–51	0.82	0.44–1.52
Household CP is an adult	25/64	39	28–51	Ref	
**PC is a child (n = 22)**	23/59	39	28–52	1.17	0.66–2.09
Household CP is a child	4/15	27	11–52	NA	NA
Household CP is an adult	19/44	43	30–58	NA	NA
**PC is an adult (n = 16)**	11/33	33	20–50	Ref	
Household CP is a child	5/13	38	18–64	NA	NA
Household CP is an adult	6/20	30	15–52	NA	NA

CI: confidence interval; CP: contact person; n: number; N: total number; NA: not applicable; PC: primary case; Ref: reference; RR: relative risk; SAR: secondary attack rates; SARS-CoV-2: severe acute respiratory syndrome coronavirus 2.

## Discussion

These simultaneously occurring outbreaks in childcare facilities provided an opportunity to revisit definitions of ‘closeness’. For pre-VOC SARS-CoV-2 strains, close (high-risk) contacts were associated with a SAR of 5% [7] to 13% [8], while non-close (or low-risk) contacts were associated with a SAR of 0% [7] to 3% [8]. At least in the childcare centre 1 and 2 outbreaks described here, contact persons not fulfilling the existing definition of a ‘close contact’ had a substantially higher SAR than previously reported. Moreover, in all three outbreaks, we documented case-clustering within approximately 1 week, which is compatible with a one- or two-day exposure for the entire childcare centre. Remarkably, transmission occurred in similar proportions to children and adults alike. In addition, transmission affected 11 of 12 groups, including the rather separated daycare groups.

During our investigation, we gained a good overview of attitudes and practices of educators and the adherence to hygiene and infection control measures in the three childcare centres. These insights enabled us to classify children in the two groups of childcare centre 3 as close contacts since we learned during the visits that they took their meals in the same room even though separated by a bookshelf and within 1.5 m distance. By visiting each centre, we observed that the space available and type of ventilation differed, which may have facilitated airborne transmission to different degrees, resulting in varied SAR between the centres; rooms in childcare centre 1 were small with low ceilings, childcare centre 2 had a ventilation system and the two groups in childcare centre 3 were spread out over two floors. In addition, childcare centre 3 was closed entirely faster than the other centres which might further explain its smaller outbreak size.

Despite the noteworthy variability of SAR between the facilities we also analysed susceptibility to the B.1.1.7 variant by broad age categories. With the exception of the childcare centre 1 outbreak, SAR were similar among children and adults within the centres. Reinforcing this point, they were also similar in the household outbreaks. This seems to imply a similar susceptibility of adults and children and marks a change to the time before the circulation of the B.1.7.7 variant, when children were associated with lower susceptibility compared with adults [9-11]. Further, the point estimates of household SAR of both children (32%; 95% CI: 18–51) and adults (39%; 95% CI: 28–51) were higher than those calculated for the pre-B.1.7.7 variant period in the meta-analysis by Madewell et al. (17%; 95% CI: 12–22 and 28%; 95% CI: 20–37, respectively) [11], which suggests an overall increase in transmissibility. However, we must concede that there is a high variability among the studies included and the CIs after meta-analysis overlap with the ones reported here.

Evidence from international studies from outbreak or household investigations involving the SARS-CoV-2 B.1.1.7 variant is limited. Data from the United Kingdom on SAR among B.1.1.7 close contacts (definition for close contacts being similar to the definition used in this study) stratified by 10-year age groups, indicate a similar relative increase among most age groups [5]. However, reported SARs among 0–9-year-olds are still lower compared with adults (9% vs ca 20%).

Important questions of our investigation were, if children – after having been infected in childcare facilities – cause further infections in their household, and if so, to what extent children infect adults in their households. The above mentioned meta-analysis of pre-VOC studies found a (non-significantly) lower SAR when children aged < 18 years were the PC (7.9%; 95% CI: 1.7–16.8) compared with SAR when adults were the household PC (15.2%; 95% CI: 6.2–27.4) [11]. In our study, we found SAR of 39% (95% CI: 28–52) for preschool child PCs and 33% (95% CI: 20–50) for adult PCs. A Danish study analysed administrative data comprising the full population [6]. SAR in households with a case infected by SARS-CoV-2 B.1.1.7 variant was found to be 38% compared to 27% in households with cases infected by other variants. A display by age groups showed a U-shaped curve for both B.1.1.7 susceptibility and infectiousness, shifted upwards from non-B.1.1.7 variants, with a low SAR for teenagers, and comparable SAR values for 0–4 and 5–9 year-old children and adults. Thus, their results compared well with ours.

We acknowledge the following limitations: first, all imperfections associated with observational data apply to our study, such as non-randomisation of childcare facilities, PCs, exposed persons and measures taken. Second, because not everyone was tested, we may have underestimated the actual SAR. However, symptom monitoring during quarantine was in place and would have led to immediate testing, thus limiting the underestimation. Third, in households, asymptomatic persons may be the true PCs, and cases outside the family may have infected a family member independently from the assumed household’s PC. Even if these possibilities may be true in individual households, it is unlikely to distort the overall picture.

## Conclusion

In summary, our investigation supports the notion of an increased transmissibility of the SARS-CoV-2 B.1.1.7 variant. In addition, the data presented suggest that both susceptibility and infectiousness of children aged between 1 to 6 years are substantially higher compared with the pre-VOC period, and may be converging to those among adults. To prevent individual childcare facility outbreaks, or at least limit outbreak size, measures in place need to be revisited, including non-pharmaceutical measures. Early closure should be considered when cases are occurring, and vaccination of staff should be promoted.
